# MAP4K4: an emerging therapeutic target in cancer

**DOI:** 10.1186/s13578-016-0121-7

**Published:** 2016-10-28

**Authors:** Xuan Gao, Chenxi Gao, Guoxiang Liu, Jing Hu

**Affiliations:** 1 Department of Respiratory Medicine, Southwest Hospital, Third Military Medical University, Chongqing, China; 2 Department of Pharmacology and Chemical Biology, University of Pittsburgh School of Medicine, Pittsburgh, USA; 3 University of Pittsburgh Cancer Institute, University of Pittsburgh School of Medicine, Hillman Cancer Center Research Pavilion, 2.42D, 5117 Centre Avenue, Pittsburgh, PA 15213 USA

**Keywords:** MAP4K4, Cancer, Therapeutic target, Signaling pathways

## Abstract

The serine/threonine kinase MAP4K4 is a member of the Ste20p (sterile 20 protein) family. MAP4K4 was initially discovered in 1995 as a key kinase in the mating pathway in *Saccharomyces cerevisiae* and was later found to be involved in many aspects of cell functions and many biological and pathological processes. The role of MAP4K4 in immunity, inflammation, metabolic and cardiovascular disease has been recognized. Information regarding MAP4K4 in cancers is extremely limited, but increasing evidence suggests that MAP4K4 also plays an important role in cancer and MAP4K4 may represent a novel actionable cancer therapeutic target. This review summarizes our current understanding of MAP4K4 regulation and MAP4K4 in cancer. MAP4K4-specific inhibitors have been recently developed. We hope that this review article would advocate more basic and preclinical research on MAP4K4 in cancer, which could ultimately provide biological and mechanistic justifications for preclinical and clinical test of MAP4K4 inhibitor in cancer patients.

## Background

MAP4K4, also known as HGK (hematopoietic progenitor kinase/germinal center kinase-like kinase) or NIK (Nck interacting kinase, the mouse ortholog) is a serine/threonine (S/T) kinase that belongs to the mammalian family of Ste20 protein kinases due to their shared homology to the budding yeast kinase Ste20p [1]. Ste20 family consists of more than 30 members that can be divided into two subgroups based on the location of their catalytic domains (N-terminals vs. C-terminals): the p21-activated kinases (PAKs, C-terminals) and the germinal center-like kinases (GCKs, N-terminals) [1]. Based on the wide variety of structure in the noncatalytic regions of the GCKS, these kinases are further divided into eight subfamilies [2]. MAP4K4 is one of four members of the mammalian GCK-IV subfamily [1, 3–6].

MAP4K4 contains ~1200 amino acids with a molecular mass of ~140 KDa [7, 8]. The orthologues of MAP4K4 among different species share similar molecular structure. Human MAP4K4 gene is located at 2q11.2 in human chrome [1]. MAP4K4 is expressed in all tissue types examined [7] but appears to express at relatively higher levels in the brain and testis [9]. Five alternatively spliced transcript variants encoding different isoforms of human MAP4K4 can be found in NCBI database. These splice variants contain identical kinase domain at the N-terminus and alternative splicing appears to mainly affect the intermediate regions of MAP4K4. Mouse MAP4K4 (NIK) has two proline-rich motifs in its intermediate domain through which NIK binds with SH3 (the SRC homology 3) domain of NCK adapter protein [8, 10]. The long version of MAP4K4 and MAP4K4 cloned from tumor cells, but not the short version of MAP4K4, also contain proline-rich regions [9]. Although the biological significance of all MAP4K4 isoforms remains to be determined, it is reasonable to speculate that variation in the middle domain could affect MAP4K4 interaction with other factors, resulting in different biochemical and physiological consequences. While multiple isoforms can be present in the same cell, the relative abundance of each isoform in a given cell appears to be different in a cell-type or tissue-type specific manner [9]. For instance, the shorter version of MAP4K4 is predominately expressed in human brain, liver, skeletal muscle and placenta, the longer version is more abundant in the brain [9]. The tissue-specific expression patterns of MAP4K4 isoforms could suggest that each isoform may have a distinct or tissue-specific function or the regulation of each isoform could be tissue- or cell type-specific.

As summarized in Table 1, the functional significance of MAP4K4 in biology has been firmly established based on genetic evidence from mouse models. Whole-body or endothelial-specific knockout of MAP4K4 is embryonic lethal due to impaired mesodermal and somite development and decreased migration activity of endothelial cells respectively [11, 12]. Besides its essential role in embryonic development, MAP4K4 has also been implicated in focal adhesion dynamics regulation [13], systemic inflammation [14], lung inflammation [15], type 2 diabetes [16, 17], atherosclerosis [18] and insulin sensitivity [19]. For detailed information regarding MAP4K4 in immunity/inflammation and metabolic/cardiovascular diseases, we refer the reader to two excellent reviews [20, 21]. In this review, we will discuss current understanding of MAP4K4 regulation and summarize evidence that implicates MAP4K4 in cancer.

**Table 1:** Summary of MAP4K4 knockout mouse models

Tissue/cell type	Phenotype	Ref.
Whole-body knockout	Embryonic lethality	[11]
Whole-body-inducible knockout	Reduced plasma glucose levels and enhanced insulin sensitivity	[19]
Skin conditional knockout	Aberrant wound repair and epidermal cell migration defects	[13]
T cell-specific knockout	Systemic inflammation and type 2 diabetes	[16]
Endothelial cell-specific knockout	Embryonic lethality	[12]
Endothelial cell-specific inducible knockout	Protected from vascular inflammation and atherosclerosis	[18]

### Regulation of MAP4K4 kinase activity and gene expression

**Fig. 1:**
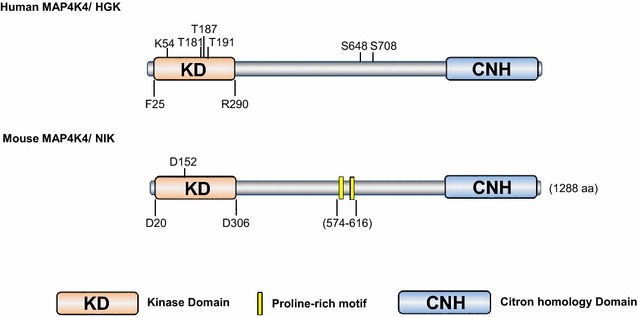
Schematic diagram of MAP4K4 structure. Both human and mouse MAP4K4 are composed of an N-terminal kinase domain and a C-terminal citron-homology domain. Mouse MAP4K4 contains proline-rich motifs. Sites involved in regulation of kinase activity and potential phosphorylation sites are indicated

Despite different localizations of their catalytic domains (N-terminus vs. C-terminus), mammalian Ste20 kinases share similar features in the kinase domain [1]. In general, activation of most Ste20 family kinases appears to require phosphorylation of a primary site in the activation segment of the kinase [1]. It is believed that phosphorylation stabilizes the activation segment in a conformation suitable for substrate binding and the unphosphorylated activation segment is largely unstructured [1]. Many Ste20 kinases also require phosphorylation of additional residues (secondary sites) by upstream kinases or from autophosphorylation for full activity [1]. In NIK, the mouse ortholog of MAP4K4, replacing aspartate (D) 152 with asparagine (N) abolished the kinase activity of NIK [8]. But potential phosphorylation site required for full kinase activity of NIK has not been identified. In human Map4K4, as summarized in Fig. 1, several amino acid residues in the N-terminal kinase domain have been implicated in the regulation of MAP4K4 activity including lysine 54 (K54), aspartate 153 (D153, corresponding site of NIK D152), threonine 181 (T181), threonine 187 (T187) and threonine 191 (T191) [9, 12, 13]. Result of in vitro kinase assay using MAP4K4 mutant protein as enzyme and myelin basic protein (MBP) as substrate showed that mutation of T187 to E slightly increased the catalytic activity of MAP4K4 [9], suggesting that this is a potential phosphorylation site. Regarding T181, there is no in vitro kinase assay result available showing that T181A (replacing threonine to alanine) mutation abolishes MAP4K4 kinase activity, whereas T181D or T181E mutation increases or restores MAP4K4 activity. However, this mutant (T181E) was used in a recent study as a phosphor-mimetic mutant [12]. Mutation of T191 to glutamate (E) or K54 to arginine (R), completely abrogated the kinase activity of MAP4K4, indicating that these two residues are required for MAP4K4 kinase activity. If T191 is a phosphorylation site, the result suggests that phosphorylation of T191 has a negative impact on MAP4K4 kinase activity. Phosphorylation of T181 and T187 or T191 has not been verified in vivo. The biochemical and biological consequences of these phosphorylations remain to be determined. Identifying upstream kinases responsible for the phosphorylation will help to understand how MAP4K4 is regulated in biological contests.

Taking the global phosphoproteomics approach and by comparing the corresponding SILAC (Stable Isotope Labeling by Amino acids in Cell) ratios of EGF (epidermal growth factor) stimulation and erlotinib (EGFR inhibitor) treatment in lung adenocarcinoma cells, a prior study identified two serine sites S648 and S708 in the middle domain of MAP4K4 as EGFR (epidermal growth factor receptor) signaling-dependent phosphorylation sites [22]. Serine 648 is conserved among the five MAP4K4 isoforms mentioned above [7, 9], but serine 708 is missing in the corresponding position of the above isoforms, suggesting that there could be an unidentified isoform of MAP4K4 or it was simply due to incorrect matches [23]. Although phosphorylation of these sites need to be further verified by mutation strategy and the biochemical and biological consequences of these phosphorylations remain to be determined, the observation that in vivo phosphorylation of these sites are regulated by EGFR signaling strongly support that the intermediate regions of MAP4K4 may also play an important role in the regulation of MAP4K4 activity or function.

The C-terminal domain of MAP4K4 contains a citron-homology domain (CNH) that appears to determine MAP4K4 association with other factors [9]. For instance, MAP4K4 interaction with Rap2 requires the entire CNH domain [24]. A human guanylate-binding protein (GBP) hGBP3, binds to the C-terminal regulatory domain of MAP4K4 [25]. Presumably through affecting protein–protein interaction, the C-terminal domain of MAP4K4 is believed to be involved in the regulation of MAP4K4 activity. It has been shown that full activation of SAPK (Stress-activated protein kinases, also known as Jun amino-terminal kinases, JNK) by MAP4K4 requires both MAP4K4′s kinase activity and the C-terminal regulatory domain that mediates the association of MAP4K4 with MEKK1 (mitogen-activated protein kinase kinase kinase 1) [8]. Although protein–protein interaction appears to determine MAP4K4 kinase activity, MAP4K4 interaction with other proteins appears to not require its kinase activity. The results of coimmunoprecipitation assay have shown that wild-type MAP4K4 and kinase-inactive MAP4K4 (MAP4K4-K54R) exerted similar binding affinity to transcription factor STAT3 (signal transducer and activator of transcription 3) in human embryonic kidney (HEK) 293T cells [9]. Consistent with this, MAP4K4 interacts with PYK2 (proline-rich tyrosine kinase 2) through the C-terminal portion of MAP4K4 and the association does not require catalytic activity of MAP4K4 [26].

Taken together, current evidence, as summarized above, strongly supports that the MAP4K4 kinase activity can be positively or negatively regulated by upstream kinases. The identities of the kinases remain largely unexplored. If multiple kinases are involved, it is highly likely that the selection within a repertoire of candidate kinases is context-dependent, depending on the cell type, the nature of the external stimuli, and the cell state. The biochemical and biological consequences of phosphorylation could also be context-dependent. In addition to negatively or positively regulating MAP4K4 kinase activity, phosphorylation may also determine MAP4K4 subcellular localization and substrate-selection.

A recent study points to a possibility that in T cells of type 2 diabetes patients, mRNA level of MAP4K4 might be affected by enhanced methylation of CpG islands in its promoter region [17], suggesting epigenetic regulation could play a role in the regulation of MAP4K4 expression. Information regarding regulation of MAP4K4 by natural stimuli and transcription factors is extremely limited. To date, only two factors have been reported to be involved in modulating MAP4K4 expression: TNF-α and p53. TNF-α treatment, through TNF-α receptor 1 (TNFR1), increases MAP4K4 mRNA and protein expression in cultured adipocytes [27]. TNF-α can stimulate MAP4K4 kinase activity in 293T cells [7] and in rat primary beta cells [28], which appears not to involve changes in MAP4K4 expression, suggesting mechanisms underlying TNF-α regulation of MAP4K4 is context-dependent. *MAP4K4* gene contains a nearby p53 binding sites cluster downstream of the promoter and six potential p53 binding sites in the first intron, four of which are confirmed by chromatin immunoprecipitation (ChIP) experiments [9, 29]. Induction of p53 in p53-Saos-2 cells upregulates MAP4K4 mRNA expression [29]. The physiological relevance of TNF-α- and p53-mediated regulation of MAP4K4 expression still needs to be verified in biological systems, given the fact that both TNF-α and p53 are broadly involved in human biology and diseases, these findings strongly support the notion that modulation of MAP4K4 expression could be an important mechanism of MAP4K4 regulation and could have important biological and clinical significance.

### MAP4K4 in cancer

Despite the fact that evidence from genetic studies using mouse model is still lacking, emerging evidence from preclinical and patient association studies strongly suggests that MAP4K4 may play an important role in many types of cancer and could serve as a novel actionable target for cancer treatment. The first evidence suggesting a role of MAP4K4 in cancer came from observations that MAP4K4 is highly expressed in 40 of the NCI-60 human tumor cell lines and can modulate cellular transformation, adhesion and invasion [9]. In this study, using MAP4K4 kinase-active (T187E) mutant or kinase-inactive mutants (K54R or T191E) as a tool and rodent cells (NIH3T3 and RIE-1 cells) as cellular models, Wright et al. found that MAP4K4, in a kinase activity-dependent manner, positively regulate cell transformation and invasion and negatively regulates cell spreading and adhesion, which provides the first clue suggesting that MAP4K4 may promote tumor development and progression. As summarized in Table 2, since 2003, there is increasing evidence pointing to the possibility that MAP4K4 plays an important role in many types of tumors. Negative association between MAP4K4 expression and patient prognosis has been observed in several types of human cancer. Current evidence indicates that MAP4K4 can potentially serve as a negative prognostic indicator in patients with colorectal cancer (CRC) [30], hepatocellular carcinoma (HCC) [31], pancreatic ductal adenocarcinoma (PDAC) [32], lung adenocarcinoma [33] and prostate cancer [34]. In cancer cells, in general, downregulation of MAP4K4 results in induction of apoptosis [31, 35–38], cell cycle arrest [31, 37, 38] and inhibition of cell growth and proliferation [31, 37, 38], migration and invasion [26, 37–40]. MAP4K4 may very likely participate in the regulation of other cellular functions and processes.

**Table 2:** Current information on MAP4K4 in cancer

Tumor type/cell line studied	Patient prognosis correlation	Manipulation of MAP4K4			Year of publication	Ref.
		Method	Functional consequences	Suggested downstream effector		
Tumors of the digestive system						
Colorectal cancer tissues and cell line	MAP4K4 expression was reversely correlated with overall survival and lymph node metastasis	NA	NA	NA	2010	[30]
Colorectal cancer tissues and cell line	NA	siRNA knockdown	Cell proliferation↓, colony formation↓, G0/G1 arrest and apoptosis↑	MAPK/JNK, MDM2	2015	[41]
Gastric cancer cells	NA	siRNA knockdown	cell proliferation↓, G1 phase arrest, apoptosis↑ and invasion↓	Notch2, Notch3, Hes1	2015	[38]
Hepatocellular carcinoma	MAP4K4 expression was a negative predictor of overall survival and early recurrence rate	shRNA knockdown	S phase arrest, apoptosis↑	MAPK/JNK, NF-κB, TLRs	2011	[31]
Hepatocellular carcinoma cell line	NA	siRNA knockdown	Cell invasion↓	MMP-2, MMP-9, NF-κB	2010	[40]
Pancreatic cancer tissues and cell lines	NA	siRNA knockdown	Cell proliferation↓, colony formation↓, invasion↓, G1 arrest, apoptosis↓, chemosensitivity↑ and xenograft tumor growth↓	NA	2013	[37]
Stage II pancreatic ductal adenocarcinoma	MAP4K4 expression was associated with poor overall and recurrence-free survival	NA	NA	NA	2008	[32]
Head and neck cancer						
Larynx carcinoma cell line	NA	siRNA knockdown	Cell migration↓ and invasion↓	JNK	2015	[35]
Lung cancer						
Lung adenocarcinoma	MAP4K4 expression was associated with shorter overall and recurrence-free survival	NA	NA	NA	2012	[33]
Lung adenocarcinoma cell lines	NA	shRNA knockdown	Cell apoptosis↑	Survivin	2014	[36]
Gynecologic cancer						
Ovarian carcinoma cell line	NA	siRNA knockdown	Cell motility↓	MAPK/JNK	2006	[39]
Genitourinary cancer						
Prostate cancer	MAP4K4 was associated with time to biochemical failure	NA	NA	NA	2014	[34]
Tumor of the central nervous system						
Glioblastoma cell line	NA	shRNA knockdown	Cell migration↓ and invasion↓	NA	2013	[26]
Mesenchymal tumor						
Kaposi’s sarcoma	NA	siRNA knockdown	Reactivation of Kaposi’s sarcoma herpesvirus and cell invasion↓	COX-2, MMP-7, MMP-13	2013	[42]
Others						
60 cell lines from NCI tumor panel	NA	Kinase-inactive mutation	Anchorage-independent growth↓, cell invasion↓, cell adhesion↑ and spreading rates↑	STAT3	2003	[9]

*NA* not available

**Fig. 2:**
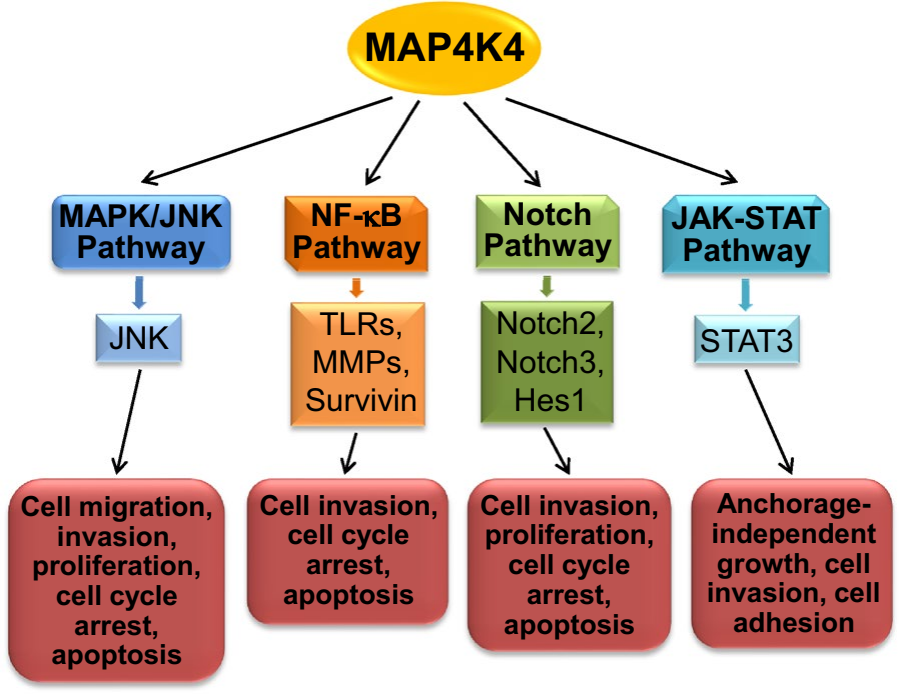
Schematic diagram of suggested MAP4K4 downstream effectors and biological outcomes in cancer. Studies on MAP4K4 in cancer have shown that MAP4K4 regulates different biological outcomes (*red boxes*) trough different cell signaling pathways (*boxes with different colors*), implicating that MAP4K4 exerts its role in regulation of tumorigenesis and tumor progression in a biological context-dependent manner

Little information exists regarding how MAP4K4 is involved in cancer. As shown in Fig. 2, knockdown of MAP4K4 affected the expression, activity or function of many factors that could act as a downstream effector or signaling mediator of MAP4K4. These factors can be grouped in several categories: kinase (MAPK/JNK) [31, 35, 39, 41]; transcription factor (NF-κB, STAT3 and HES1) [9, 31, 38, 40]; transmembrane receptor important for cell–cell communication (Notch2 and Notch3) [38]; matrix metalloproteinases (MMP, MMP-2, MMP-9, MMP-7 and MMP13) [40, 42]; inhibitor of apoptosis negative regulator of the p53 tumor suppressor (MDM2) [41] and inflammation related factor (cyclooxygenase-2 and toll-like receptors) [31, 42]. Among the above-mentioned pathways or factors, it is interesting to note that in most studies, MAP4K4 exerts its function not through canonical MAPK pathways as expected. The nomenclature of Ste20 kinases as MAP4Ks was based on their regulation of MAPK pathways through activating MAP3Ks [2]. The first evidence of MAP4K4 regulation of MAPK/JNK came from a study showing that co-expression of kinase-defective MEKK1 and MAP4K4 in cultured adipocytes inhibited the activation of JNK by MAP4K4 [8]. Consistent with this, a later study found that dominant-negative mutant of TAK1, an MAP3 K, significantly inhibited MAP4K4-induced JNK activation [7]. Besides MAPK/JNK, MAP4K4 has also been reported to regulate MAPK/ERK1/2 pathway and MAPK/p38 pathway in biological systems other than cancer [43–46]. Together, current findings suggest that MAP4K4 may contribute to cancer mainly through canonical MAPK-independent mechanisms.

In addition to above mentioned candidate downstream mediators of MAP4K4, MAP4K4 also participated in the regulation of other cancer-related signaling pathways or factors including insulin pathway, hippo signaling (LATS1/2 and YAP/TAZ) and mTOR/AMPK [19, 21, 43, 47–52]. Although experimental evidence that supports the link between MAP4K4 and these pathways and cancer is currently not available, it is reasonable to believe that MAP4K4 could contribute to cancer through modulating these pathways or factors in a context-dependent manner.

How MAP4K4 regulates its downstream effectors or signaling mediators remains largely unexplored. Since MAP4K4 is a kinase, one would expect that the primary job of MAP4K4 is to phosphorylate its substrate. Indeed, MAP4K4 can directly phosphorylate TRAF2 at serine 35 to promote its degradation in T cells [16]. However, none of these factors have been proved to be a direct phosphorylation substrate of MAP4K4 in cancer, implying that MAP4K4 regulates these factors or pathways indirectly. Identification of direct substrates of MAP4K4 will provide crucial clues as to how MAP4K4 is mechanistically involved in cancer.

As summarized in Table 3, several MAP4K4-specific small-molecule inhibitors are currently available. Some of the inhibitors show promise in treating plaque development, pathological angiogenesis in mice [12, 18, 53–55]. Whether these inhibitors possess potent antitumor properties remains to be determined. Given the potential role of MAP4K4 in cancer, we believe MAP4K4 inhibition may be a possible new direction in cancer therapy.

**Table 3:** MAP4K4-specific small-molecule inhibitors

Name	Type	Specificity	Biological/Preclinical disease model	Clinical trial	Ref.
GNE-495	KI	MAP4K4	Angiogenesis in mouse model	NA	[12, 53]
GNE-220	KI	MAP4K4	Angiogenesis in mouse model	NA	[12]
PF-6260933	KI	MAP4K4	Vascular inflammation, atherosclerosis	NA	[18, 54]
4-Hydroxy-2-pyridone	KI	MAP4K4	Neuroblastoma cell	NA	[55]

*KI* kinase inhibitor, *NA* not available

### Perspective

Current evidence supports but not yet provides sufficient biological and mechanistic justification for MAP4K4 as a novel cancer therapeutic target. Evidence definitely linking MAP4K4 to the development and progression of any types of cancer is still lacking. To this end, it is essential to examine the impact of genetic manipulation of MAP4K4 in mouse models of cancer. When interpreting results from experiments using MAP4K4 knockout mice (whole-body or tissue-specific knockout), potential redundancy and functional compensation among MAP4Ks should be taken into consideration. Since small-molecule inhibitors of MAP4K4 are available, in order to eventually use these inhibitors in clinic, future studies should attempt to test their tumor prevention and antitumor activity in mouse models of cancer.

Currently there is no sufficient information suggesting in which type of cancer MAP4K4 inhibitor can be used as a novel promising therapy. Target overexpression is an overrated predictor of efficacy since it may also represent a cellular attempt to limit unbridled growth, unless functional results from genetic manipulation of MAP4K4 in that particular tumor model are available, it is difficult to predict cancer types responsive to MAP4K4 inhibitor treatment. It is possible that MAP4K4 could promote tumor development and progression in certain types of cancer and functions as a tumor repressor in other types of cancer, or plays different roles at different stages during tumor development and progression. Therefore it is crucial to gain a thorough understanding of how MAP4K4 is involved in a particular type of cancer functionally and mechanistically before conducting clinical testing of MAP4K4 inhibitor in cancer patients.

No information is available about whether and how MAP4K4 is involved in resistance to standard cancer therapy. If MAP4K4 contributes to cancer development and progression, it is highly likely that MAP4K4 can also be involved in treatment resistance. Therefore in addition to examine the potential anti-tumor activity of MAP4K4 inhibitors as a standalone therapy, it is also important to test if MAP4K4 inhibitors can be used in combination to overcome resistance to chemotherapy, radiation therapy, targeted therapy and immunotherapy.

Detailed molecular understanding of how MAP4K4 is involved in cancer biology is essential for firmly establishing MAP4K4 as a target for that particular type of cancer. Crucial to this effort is to identify key upstream regulators and downstream effectors including substrates of MAP4K4. To develop efficient methods to block MAP4K4, it is also crucial to understand how MAP4K4 functionally interacts with Ste20 family members.

Cancer remains a largely incurable disease, indicating an urgent and unmet need for novel effective therapeutic approaches. Identifying a novel cancer therapeutic target that could be amenable to pharmacologic intervention is challenging. To this end, we believe that a better understanding of biological functions and underlying mechanisms of MAP4K4 in cancer could have far-reaching implications for new directions in cancer therapy.

## Abbreviations

ChIP: chromatin immunoprecipitation
EGF: epidermal growth factor
HGK: hematopoietic progenitor kinase/germinal center kinase-like kinase
JNK: Jun amino-terminal kinases
MAP4K4: mitogen-activated protein kinase kinase kinase kinase 4
MMP: matrix metalloproteinases
NIK: Nck interacting kinase
PYK2: proline-rich tyrosine kinase 2
shRNA: short hairpin RNA
STAT3: signal transducer and activator of transcription 3

## References

[1] Delpire E (2009). The mammalian family of sterile 20p-like protein kinases. Pflugers Arch.

[2] Dan I, Watanabe NM, Kusumi A (2001). The Ste20 group kinases as regulators of MAP kinase cascades. Trends Cell Biol.

[3] Dan I (2000). Molecular cloning of MINK, a novel member of mammalian GCK family kinases, which is up-regulated during postnatal mouse cerebral development. FEBS Lett.

[4] Fu CA (1999). TNIK, a novel member of the germinal center kinase family that activates the c-Jun N-terminal kinase pathway and regulates the cytoskeleton. J Biol Chem.

[5] Kanai-Azuma M (1999). Nrk: a murine X-linked NIK (Nck-interacting kinase)-related kinase gene expressed in skeletal muscle. Mech Dev.

[6] Nakano K (2000). NESK, a member of the germinal center kinase family that activates the c-Jun N-terminal kinase pathway and is expressed during the late stages of embryogenesis. J Biol Chem.

[7] Yao Z (1999). A novel human STE20-related protein kinase, HGK, that specifically activates the c-Jun N-terminal kinase signaling pathway. J Biol Chem.

[8] Su Y-C (1997). NIK is a new Ste20-related kinase that binds NCK and MEKK1 and activates the SAPK/JNK cascade via a conserved regulatory domain. EMBO J.

[9] Wright JH (2003). The STE20 kinase HGK is broadly expressed in human tumor cells and can modulate cellular transformation, invasion, and adhesion. Mol Cell Biol.

[10] Becker E (2000). Nck-interacting Ste20 kinase couples Eph receptors to c-Jun N-terminal kinase and integrin activation. Mol Cell Biol.

[11] Xue Y (2001). Mesodermal patterning defect in mice lacking the Ste20 NCK interacting kinase (NIK). Development.

[12] Vitorino P (2015). MAP4K4 regulates integrin-FERM binding to control endothelial cell motility. Nature.

[13] Yue J (2014). Microtubules regulate focal adhesion dynamics through MAP4K4. Dev Cell.

[14] Aouadi M (2009). Orally delivered siRNA targeting macrophage Map4k4 suppresses systemic inflammation. Nature.

[15] Jin M (2016). MAP4K4 deficiency in CD4(+) T cells aggravates lung damage induced by ozone-oxidized black carbon particles. Environ Toxicol Pharmacol.

[16] Chuang HC (2014). HGK/MAP4K4 deficiency induces TRAF2 stabilization and Th17 differentiation leading to insulin resistance. Nat Commun.

[17] Chuang H-C (2016). Epigenetic regulation of HGK/MAP4K4 in T cells of type 2 diabetes patients. Oncotarget.

[18] Roth Flach RJ (2015). Endothelial protein kinase MAP4K4 promotes vascular inflammation and atherosclerosis. Nat Commun.

[19] Danai LV (2015). Inducible deletion of protein kinase Map4k4 in obese mice improves insulin sensitivity in liver and adipose tissues. Mol Cell Biol.

[20] Chuang HC, Wang X, Tan TH (2016). MAP4 K family kinases in immunity and inflammation. Adv Immunol.

[21] Virbasius JV, Czech MP (2016). Map4k4 signaling nodes in metabolic and cardiovascular diseases. Trends Endocrinol Metab.

[22] Zhang X (2015). Identifying novel targets of oncogenic EGF receptor signaling in lung cancer through global phosphoproteomics. Proteomics.

[23] Dephoure N (2013). Mapping and analysis of phosphorylation sites: a quick guide for cell biologists. Mol Biol Cell.

[24] Machida N (2004). Mitogen-activated protein kinase kinase kinase kinase 4 as a putative effector of Rap2 to activate the c-Jun N-terminal kinase. J Biol Chem.

[25] Luan Z (2002). A novel GTP-binding protein hGBP3 interacts with NIK/HGK. FEBS Lett.

[26] Loftus JC (2013). A Novel Interaction between Pyk2 and MAP4K4 Is integrated with glioma cell migration. J Signal Transduct.

[27] Tesz GJ (2007). Tumor necrosis factor alpha (TNFα) stimulates Map4k4 expression through TNFα receptor 1 signaling to c-Jun and activating transcription factor 2. J Biol Chem.

[28] Bouzakri K, Ribaux P, Halban PA (2009). Silencing mitogen-activated protein 4 kinase 4 (MAP4K4) protects beta cells from tumor necrosis factor-alpha-induced decrease of IRS-2 and inhibition of glucose-stimulated insulin secretion. J Biol Chem.

[29] Miled C (2005). A genomic map of p53 binding sites identifies novel p53 targets involved in an apoptotic network. Cancer Res.

[30] Hao JM (2010). A five-gene signature as a potential predictor of metastasis and survival in colorectal cancer. J Pathol.

[31] Liu AW (2011). ShRNA-targeted MAP4K4 inhibits hepatocellular carcinoma growth. Clin Cancer Res.

[32] Liang JJ (2008). Expression of MAP4K4 is associated with worse prognosis in patients with stage II pancreatic ductal adenocarcinoma. Clin Cancer Res.

[33] Qiu MH (2012). Expression and prognostic significance of MAP4K4 in lung adenocarcinoma. Pathol Res Pract.

[34] Rizzardi AE (2014). Evaluation of protein biomarkers of prostate cancer aggressiveness. BMC Cancer.

[35] Yang N (2015). Silencing SOX2 expression by rna interference inhibits proliferation, invasion and metastasis, and induces apoptosis through MAP4K4/JNK signaling pathway in human laryngeal cancer TU212 cells. J Histochem Cytochem.

[36] Chen S (2014). SOX2 regulates apoptosis through MAP4K4-survivin signaling pathway in human lung cancer cells. Carcinogenesis.

[37] Zhao G (2013). miRNA-141, downregulated in pancreatic cancer, inhibits cell proliferation and invasion by directly targeting MAP4K4. Mol Cancer Ther.

[38] Liu YF (2015). Silencing of MAP4K4 by short hairpin RNA suppresses proliferation, induces G1 cell cycle arrest and induces apoptosis in gastric cancer cells. Mol Med Rep..

[39] Collins CS (2006). A small interfering RNA screen for modulators of tumor cell motility identifies MAP4K4 as a promigratory kinase. Proc Natl Acad Sci USA.

[40] Han S-X (2010). Lowered HGK expression inhibits cell invasion and adhesion in hepatocellular carcinoma cell line HepG2. World J Gastroenterol.

[41] Wang B (2015). MiR-194, commonly repressed in colorectal cancer, suppresses tumor growth by regulating the MAP4K4/c-Jun/MDM2 signaling pathway. Cell Cycle.

[42] Haas DA (2013). The inflammatory kinase MAP4K4 promotes reactivation of Kaposi’s sarcoma herpesvirus and enhances the invasiveness of infected endothelial cells. PLoS Pathog.

[43] Bouzakri K, Zierath JR (2007). MAP4K4 gene silencing in human skeletal muscle prevents tumor necrosis factor-alpha-induced insulin resistance. J Biol Chem.

[44] Huang H (2014). MAP4K4 deletion inhibits proliferation and activation of CD4(+) T cell and promotes T regulatory cell generation in vitro. Cell Immunol.

[45] Tan X (2015). Cellular microRNA Let-7a suppresses KSHV replication through targeting MAP4K4 signaling pathways. PLoS ONE.

[46] Zohn IE (2006). p38 and a p38-interacting protein are critical for downregulation of E-cadherin during mouse gastrulation. Cell.

[47] Tang X (2006). An RNA interference-based screen identifies MAP4K4/NIK as a negative regulator of PPARgamma, adipogenesis, and insulin-responsive hexose transport. Proc Natl Acad Sci USA.

[48] Zhao X (2012). microRNA-30d induces insulin transcription factor MafA and insulin production by targeting mitogen-activated protein 4 kinase 4 (MAP4K4) in pancreatic beta-cells. J Biol Chem.

[49] Wang M (2013). Identification of Map4k4 as a novel suppressor of skeletal muscle differentiation. Mol Cell Biol.

[50] Hansen CG, Moroishi T, Guan KL (2015). YAP and TAZ: a nexus for Hippo signaling and beyond. Trends Cell Biol.

[51] Meng Z (2015). MAP4 K family kinases act in parallel to MST1/2 to activate LATS1/2 in the Hippo pathway. Nat Commun.

[52] Danai LV (2013). Map4k4 suppresses Srebp-1 and adipocyte lipogenesis independent of JNK signaling. J Lipid Res.

[53] Ndubaku CO (2015). Structure-based design of GNE-495, a potent and selective map4k4 inhibitor with efficacy in retinal angiogenesis. ACS Med Chem Lett.

[54] Ammirati M (2015). Discovery of an invivo tool to establish proof-of-concept for map4k4-based antidiabetic treatment. ACS Med Chem Lett.

[55] Schroder P (2015). Neuritogenic militarinone-inspired 4-hydroxypyridones target the stress pathway kinase MAP4K4. Angew Chem Int Ed Engl.

